# *Escherichia coli hns* and methylglyoxal link gut microbial metabolism to host neuronal gene regulation in Parkinson’s disease

**DOI:** 10.1080/19490976.2026.2739674

**Published:** 2026-10-15

**Authors:** Min-Gi Shin, Soo Jung Park, Woorim Kang, Yiseul Bae, Suyeon Kim, Dayeong Yoo, Seoyeon Yoon, Sang Myun Park, Eun-Soo Kwon, Jaerak Chang, Jung Han Yoon, Don Gueu Park, Eun Jeong Lee

**Affiliations:** a Department of Brain Science, Ajou University School of Medicine, Suwon, Republic of Korea; b Department of Biological Science, Kangwon National University, Chuncheon, Republic of Korea; c Department of Biomedical Sciences, Graduate School of Ajou University, Suwon, Republic of Korea; d BK21 R&E Initiative for Advanced Precision Medicine, Ajou University School of Medicine, Suwon, Republic of Korea; e Department of Pharmacology, Ajou University School of Medicine, Suwon, Republic of Korea; f Aging Convergence Research Center, Korea Research Institute of Bioscience and Biotechnology (KRIBB), Daejeon, Republic of Korea; g Department of Neurology, Ajou University School of Medicine, Suwon, Republic of Korea

**Keywords:** Parkinson’s disease (PD), gut microbiota, *hns*, *Escherichia coli* (*E. coli*), methylglyoxal (MG), *rig-5*, *LSAMP*

## Abstract

Parkinson’s disease (PD) is a progressive neurodegenerative disorder characterized by dopaminergic neuron loss in the substantia nigra (SN). Although the gut microbiota has been implicated in PD pathogenesis, how defined bacterial genes and their metabolites influence host neuronal gene regulation remains unclear. Here, we identify a gut microbial *hns*-methylglyoxal (MG) pathway linking *Escherichia coli (E. coli)* metabolism to host neuronal gene regulation. In the stool metagenomic cohort, *hns*, a bacterial gene encoding the histone-like nucleoid structuring protein and primarily derived from *E. coli*, was enriched particularly in body-first PD, a subtype associated with gut-originating pathology. In a separate plasma MG cohort, plasma levels of MG, a reactive metabolite whose bacterial production was *hns*-dependent in our model system, were higher in body-first than in brain-first PD and were nominally inversely associated with cardiac meta-iodobenzylguanidine (MIBG) uptake within the body-first group. Using *Caenorhabditis elegans* (*C. elegans*) PD models, we found that feeding with Δ*hns E. coli* protected dopaminergic neurons, whereas exogenous MG abolished this neuroprotection. Transcriptomic and neuronal RNAi analyses identified *rig-5* as a host mediator of *hns* deletion-induced neuroprotection. Cross-species analyses identified *LSAMP* as a candidate mammalian ortholog of *rig-5*. MG suppressed *LSAMP* expression in human neuronal cells, and *LSAMP* expression was reduced in the post-mortem SN of patients with PD. Conversely, *Lsamp* expression was increased in the SN of mice colonized with Δ*hns E. coli*. Together, these findings delineate a microbial gene–metabolite–host gene pathway through which *E. coli hns* and MG modulate *rig-5*-dependent dopaminergic vulnerability in *C. elegans*, with corresponding changes of *LSAMP* expression in mammalian systems.

## Introduction

Parkinson’s disease (PD) is a progressive neurodegenerative disorder characterized by dopaminergic neuron loss in the substantia nigra (SN) and pathological accumulation of *α*-synuclein (αSyn). Rather than a single disease entity, PD is increasingly recognized as a heterogeneous disorder,^1^ and the body-first versus brain-first hypothesis provides a useful framework for understanding this heterogeneity.^2^ According to this model, body-first PD is proposed to originate in the gut and peripheral autonomic nervous system before spreading to the central nervous system, whereas brain-first PD is thought to begin within the brain.^2^ Consistent with this peripheral-to-central progression, body-first PD is frequently associated with prodromal rapid eye movement sleep behavior disorder (RBD), reduced cardiac meta-iodobenzylguanidine (MIBG) uptake, and early autonomic dysfunction.^3^

Accumulating evidence implicates the gut microbiome in PD pathogenesis.^4-6^ Within the body-first versus brain-first framework, gut microbial factors may be particularly relevant to body-first PD. However, few studies have examined the gut microbiome in the context of PD subtypes. In our previous PD subtype-specific metagenomic analysis, we found increased abundance of *Escherichia coli* (*E. coli*) and enrichment of curli genes in body-first PD patients.^7^ Given the established role of *E. coli*-derived curli in promoting αSyn pathology in experimental models,^8-10^ these findings provided patient-derived evidence linking bacterial genes to a clinically defined PD subtype.

Despite these advances, how other microbial genes and their associated metabolites relate to host regulatory pathways involved in dopaminergic vulnerability remains largely unexplored. One candidate metabolite is methylglyoxal (MG), a highly reactive glycolytic byproduct that promotes oxidative stress, protein glycation, mitochondrial dysfunction,^11^ and αSyn toxicity.^12^ MG-derived advanced glycation end-products (AGEs) accumulate in PD brains and frequently co-localize with αSyn pathology.^13^ ^,^ ^14^ Experimental studies further demonstrate that MG exacerbates dopaminergic neuron loss and motor dysfunction.^14-16^ Importantly, MG is produced not only by host cells but also by gut microbes,^17^ and microbially derived MG has recently been shown to modulate host lifespan^18^ and immune responses.^19^

We therefore asked whether *hns*-dependent bacterial MG production constitutes a microbial mechanism linking gut dysbiosis to dopaminergic vulnerability in body-first PD. Using an integrative cross-species approach combining PD subtype-specific metagenomics, plasma MG measurements, *C. elegans* genetic assays, human neuronal cells, post-mortem brain datasets, and mouse colonization experiments, we identify an *E. coli hns*–MG pathway that modulates *rig-5*-dependent dopaminergic vulnerability in *C. elegans* and is associated with changes in the expression of the candidate mammalian counterpart *LSAMP*/*Lsamp*.

## Patients/materials and methods

### Human study approval and informed consent

This study was conducted in accordance with the principles of the Declaration of Helsinki (as revised in 2013). The study protocol was reviewed and approved by the Institutional Review Board of Ajou University Hospital, Suwon, Republic of Korea (AJOUIRB-SM-2023-550). All participants provided written informed consent prior to enrollment, including consent for the collection and analysis of blood samples and clinical data.

### Classification of body-first, brain-first, and undetermined PD subtypes

PD subtyping was performed using the dual criteria of premotor RBD and cardiac ^123^I-MIBG uptake, following the approach of our previous study.^7^ Premotor RBD was defined as a positive response on the REM Sleep Behavior Disorder Single-Question Screen (RBD1Q),^20^ with dream-enactment behaviors reported more than one year before the onset of motor symptoms based on clinical history. The REM Sleep Behavior Disorder Screening Questionnaire (RBDSQ)^21^ was also administered and reported as a descriptive clinical characteristic, but was not used for subtype classification. Cardiac sympathetic denervation was assessed by ^123^I-MIBG myocardial scintigraphy with images acquired at 15 minutes (early) and 180 minutes (delayed) after tracer injection; a delayed H/M ratio below 1.70 was considered abnormal. Patients with both premotor RBD and abnormal cardiac MIBG uptake were classified as body-first; patients with neither (no premotor RBD and normal MIBG) were classified as brain-first; and patients who did not clearly meet either category were designated as undetermined. Clinical and demographic characteristics of the plasma MG cohort (Figure 2) are summarized in Table S1. Clinical characteristics of the fecal metagenomic cohort (Figure 1) were described previously.^7^

**Figure 1.**
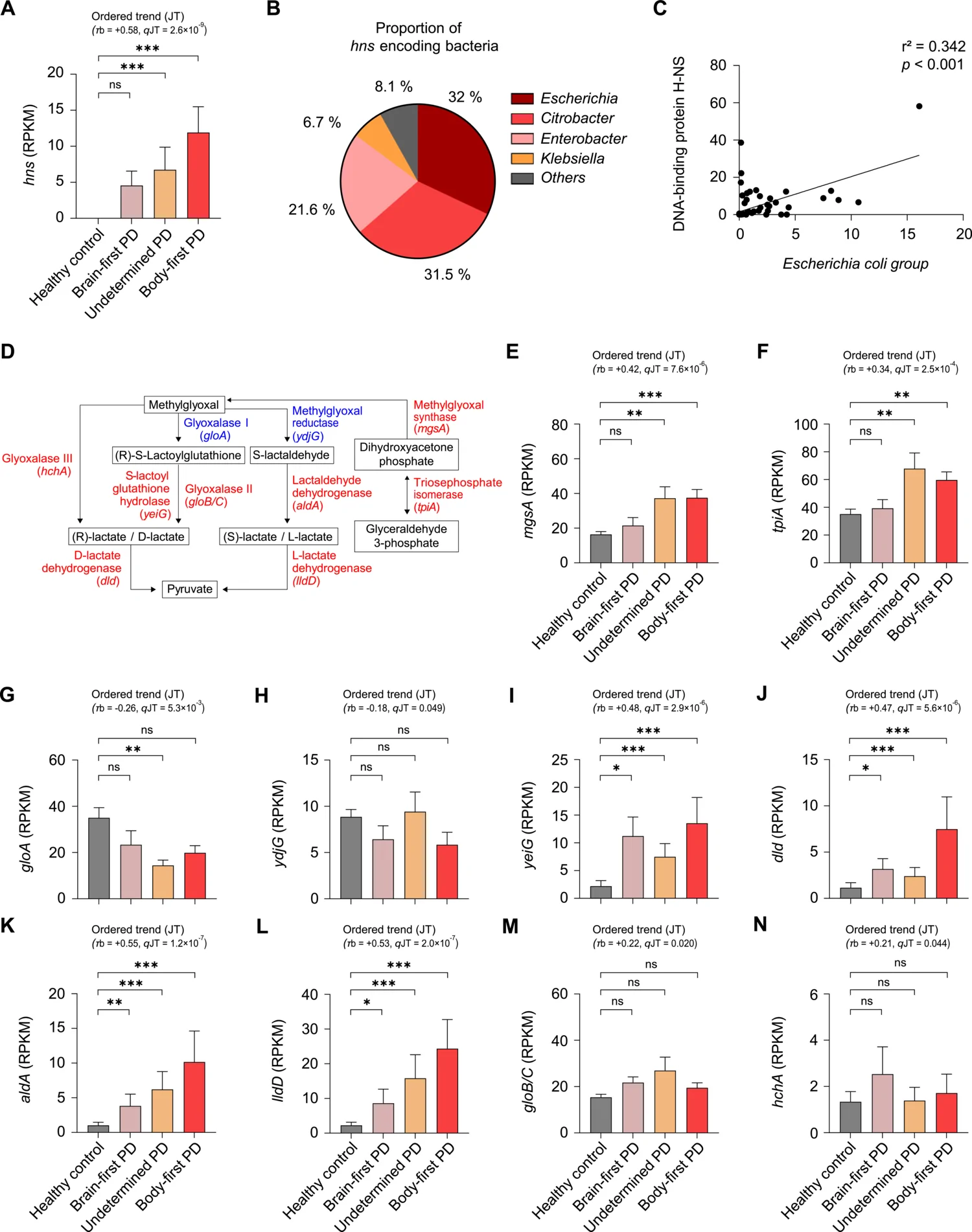
Increased gut microbial *hns* in the stool of body-first PD patients. (A) Relative abundance of *hns* in fecal samples from healthy controls and patients with body-first, brain-first, or undetermined PD. Following Bonferroni correction, *hns* abundance was significantly higher in body-first and undetermined PD than in healthy controls, whereas brain-first PD did not differ from healthy controls. Ordered-trend statistics are shown as Kendall’s tau-b and FDR-adjusted Jonckheere–Terpstra q values. (B) Composition of *hns*-encoding bacterial genera. (C) Correlation between *E. coli* relative abundance and *hns* abundance. Pearson correlation analysis was performed to assess the relationship between *E. coli* relative abundance and *hns* abundance. (D) Schematic of the bacterial MG metabolic pathway. Red and blue indicate genes showing increasing and decreasing trends, respectively, across the ordered groups from healthy controls to body-first PD, as assessed using the Jonckheere–Terpstra trend test. (E–N) Relative abundance of MG metabolic genes in fecal microbiomes of PD patients and healthy controls. Abundance of *mgsA* (Methylglyoxal synthase) (E), *tpiA* (Triosephosphate isomerase) (F), *gloA* (Glyoxalase I) (G), *ydjG* (Methylglyoxal reductase) (H), *yeiG* (S-lactoylglutathione hydrolase) (I), *dld* (D-lactate dehydrogenase) (J), *aldA* (Lactaldehyde dehydrogenase) (K), *lldD* (L-lactate dehydrogenase) (L), *gloB*/*C* (Glyoxalase II) (M), and *hchA* (Glyoxalase III) (N) is shown. Ordered-trend statistics are shown above each panel as Kendall’s tau-b and FDR-adjusted Jonckheere–Terpstra q values. Data are presented as mean ± SE. Statistical significance was determined using the two-sided Mann–Whitney U tests with Bonferroni correction within each gene for planned pairwise comparisons. Ordered trends across groups were assessed using the Jonckheere–Terpstra trend test, with FDR-adjusted q values and Kendall’s tau-b effect sizes shown in each panel. *P < 0.05, **P < 0.01, ***P < 0.001 (metagenomic cohort; *n* = 36 for healthy controls, *n* = 15 for body-first PD, *n* = 9 for brain-first PD, *n* = 12 for undetermined PD).

### Shotgun metagenomic data analysis

The analysis was conducted using the same pipeline described in Park et al. ^7^ All preprocessing steps, quality control measures, and downstream analyses were performed following the methodology outlined in the referenced study. For a detailed description of the analytical workflow, refer to Park et al. ^7^ Briefly, all fecal metagenomic samples used in Figure 1 were derived from the same previously published cohort and were processed using the same preprocessing, quality-control, taxonomic profiling, gene-family profiling, and normalization procedures. Gene abundance values were normalized as reads per kilobase per million mapped reads (RPKM) before group-level comparisons. Detection of a gene was defined operationally as a non-zero RPKM value after the same mapping and normalization procedure; therefore, low RPKM values were interpreted as low normalized abundance rather than true absence unless no mapped signal was detected. The analyses in the present study were performed within this single metagenomic dataset and did not involve merging across sequencing platforms or independent metagenomic cohorts. Detailed preprocessing, normalization, quality-control, and batch-handling procedures are provided in Park et al. ^7^ Planned pairwise comparisons for Figure 1 were analyzed using two-sided Mann–Whitney U tests with Bonferroni correction within each gene, and ordered trends across clinically defined groups were assessed using the Jonckheere–Terpstra trend test with FDR-adjusted q values and Kendall’s tau-b effect sizes.

### Plasma MG measurement by ELISA

Plasma MG levels were determined using a human MGO ELISA kit (AFG Scientific, U.S.A., EK716112) based on a quantitative sandwich ELISA principle. Briefly, samples and standards were added to a microplate pre-coated with an MGO-specific capture antibody. After binding, an HRP-conjugated detection antibody specific for MGO was added to form a sandwich complex. Unbound components were washed away, and TMB substrate was added for color development. The reaction was terminated with a stop solution, and the optical density was measured at 450 nm. All samples were analyzed in duplicate and quantified using a standard curve.

### *E. coli* strains and growth conditions

For bacteria feeding experiments, excluding RNAi-related experiments, BW25113 was used as the wild-type (WT) *E. coli*, while Δ*hns E. coli* was obtained from the *E. coli* single-gene deletion mutant library (Keio collection) with an *hns* deletion in the BW25113 background.^22^ The bacteria were inoculated in Luria-Broth (LB) medium and grown overnight at 37 °C. The cultured bacteria were then seeded onto NGM plates and incubated at room temperature for an additional day before use.

### *E. coli* lysate MG measurement by LC-MS/MS

*E. coli* lysate MG was quantified by isotope-dilution LC-MS/MS following derivatization with o-phenylenediamine (OPD), with minor modifications to previously reported methods.^23^ Bacterial pellets were resuspended in distilled water and disrupted by sonication,^18^ and protein concentrations were measured using the Pierce BCA Protein Assay Kit (Thermo Fisher Scientific, 23225). A 10 μL aliquot of lysate was mixed with 10 μL of 50 μM ^13^C₃-MG internal standard (TRC, TRC-P998872) and 180 μL of 7% perchloric acid (Sigma-Aldrich, 244252). After centrifugation, 40 μL of the supernatant was mixed with 40 μL of derivatization solution prepared using OPD (Sigma-Aldrich, P23938), HCl (Sigma-Aldrich, 258148), DETAPAC (Sigma-Aldrich, D6518), and sodium azide (Sigma-Aldrich, S2002), and incubated for 1 h at room temperature in the dark to form the stable quinoxaline derivative 2-methylquinoxaline (2-MQ). Calibration standards and quality control samples were prepared in 1% bovine serum albumin (USB Corporation, 10857) in PBS (Sigma-Aldrich, P4417) as a surrogate matrix. MG was quantified using linear calibration curves generated with an MG standard (TRC, TRC-P998870) and the ^13^C₃-MG internal standard. Concentrations were normalized to total protein content and expressed as ng/mg protein. Samples were analyzed using an Agilent 1290 Infinity II LC system coupled to a SCIEX QTRAP 5500+ mass spectrometer in positive electrospray ionization mode. Separation was performed on a ZORBAX Eclipse C18 column (3.0 × 100 mm, 3.5 μm) with 0.1% formic acid (Fluka, 56302) in water and acetonitrile (Burdick & Jackson, AH015-4A) (60:40, v/v) at 0.35 mL/min.

MRM transitions were m/z 145.1 → 77.1 for 2-MQ and m/z 148.1 → 77.1 for ^13^C₃-2-MQ.

### *C. elegans* strains and growth conditions

All experiments were initiated by placing synchronized L1 larvae onto NGM plates seeded with bacteria. The worms were maintained at 20 °C throughout the experiment. To prevent progeny proliferation, synchronized young adult worms were exposed to 0.1 mg/mL FUdR (Tokyo Chemical Industry, D2235). Synchronized L1 larvae were obtained through egg isolation followed by L1 arrest. All strains were maintained on NGM plates seeded with *E. coli* OP50 at 20 °C before egg isolation. For the 6-OHDA-induced Parkinson’s disease (PD) model, BZ555 [*dat-1p*::GFP] was used. For the αSyn-expressing PD model, UA44 [*dat-1p*::αSyn; *dat-1p*::GFP] was utilized. To perform neuronal RNAi experiments, TU3401 (*sid-1* (pk3321); uIs69 [*myo-2p*::mCherry; *unc-119p*::*sid-1*]) was crossed with BZ555 or UA44. A new strain was established by selecting progeny carrying both fluorescent markers. The UA44 strain, originally generated in Kim Caldwell’s laboratory (University of Alabama), was kindly provided by Prof. Kyung-Won Kim (Hallym University) with permission from Kim Caldwell's laboratory, while BZ555 and TU3401 were obtained from the Caenorhabditis Genetics Center (CGC). For MG supplementation experiments, 10 mM MG (Sigma-Aldrich, M0252) was added to NGM plates from the L1 stage.

## 6-OHDA treatment for PD model construction

For the 6-OHDA-induced PD model, L3-stage worms were exposed to a final concentration of 50 mM 6-OHDA (Sigma-Aldrich, H4381) prepared with 10 mM ascorbic acid (Sigma-Aldrich, A92902). After 1 hour of incubation, worms were washed three times with 1 mL of M9 buffer and then transferred onto bacteria-seeded NGM plates for subsequent experiments.

### Observation and assessment of dopaminergic neuronal morphology

Approximately 40–50 worms were carefully transferred onto a 2% agar pad on a glass slide and immobilized with 20 μL of 20 mM sodium azide in M9 buffer (Sigma-Aldrich, S2002). Worms were then examined using a LSM800 (ZEISS) confocal microscope. Neuronal morphology was manually assessed by an observer blinded to the experimental condition, and neuronal loss was categorized based on the number of missing neurons. Each worm was classified into a severity category as follows: 0–1 missing neurons (mild), 2 missing neurons (moderate), and 3–4 missing neurons (severe). Neuronal morphology was analyzed on day 3.

### RNAi knockdown

The *rig-5* RNAi vector was generated through traditional cloning methods. A 633 bp fragment was amplified using the primers FW: CTCGGTCGACGAAAAAGTGTTTCGA and RV: CTCGGAGCTCTTCTGGAATTAAATT, then inserted into the L4440 vector via SalI (TaKaRa Bio, 1080A) and SacI (TaKaRa Bio, 1078A) restriction enzyme digestion. The resulting *rig-5* RNAi construct was introduced into HT115 or Δ*hns* HT115 *E. coli* through heat shock transformation. Transformed bacteria were cultured overnight at 37 °C in LB medium containing 50 μg/mL ampicillin (Sigma-Aldrich, A0166) and 10 μg/mL tetracycline (Sigma-Aldrich, T7660). The following day, 1% of the overnight culture was transferred into fresh LB medium supplemented with 50 μg/mL ampicillin and incubated for 3 hours. The bacterial culture was then concentrated 10-fold and spread onto NGM plates supplemented with 50 μg/mL ampicillin and 1 mM IPTG (Sigma-Aldrich, I6758). Observation and assessment of dopaminergic neuronal morphology were conducted on day 3.

### Preparation of *α*-synuclein preformed fibrils (PFFs)

Recombinant human monomeric α-synuclein (rPeptide, S-1001-2) was used to prepare α-synuclein PFFs. Lyophilized *α*-synuclein was dissolved in sterile phosphate-buffered saline (PBS) to 5 mg/mL and incubated at 37 °C for 7 days with continuous agitation (2,000 × g) on an orbital shaker (Thermomixer F1.5). The resulting α-synuclein PFFs were stored at −80 °C until use. Thioflavin T binding assays confirmed fibril formation. Prior to use, the α-synuclein PFFs were briefly sonicated on ice (1 min, 20% amplitude, 1-s pulse on/off) using an ultrasonic processor (VC 505).

### Human cell line culture

SH-SY5Y cells^24^ were generously provided by Professor Sang Myun Park (Department of Pharmacology, Ajou University School of Medicine). These cells were cultured in Dulbecco's modified Eagle medium (DMEM; Gibco, 11995-065) supplemented with 10% fetal bovine serum (Sigma-Aldrich, F2442) and maintained in a humidified incubator at 37 °C with 5% CO_2_.

### Bulk RNA sequencing and analysis of human SH-SY5Y neuroblastoma cells

Total RNA was isolated from SH-SY5Y human neuroblastoma cells using an RNeasy Plus Mini Kit (Qiagen, 74134). To evaluate transcriptomic changes, cells were assigned to three groups (*n* = 3 per group): Control, MG-treated (MG) and MG plus NAC-treated (MG+NAC). MG (Sigma-Aldrich, M0252) and NAC (Sigma-Aldrich, A9165) were used. Paired-end RNA sequencing was performed on the Illumina NovaSeqX platform. Adapter trimming and quality filtering were conducted using Trimmomatic v0.38, and cleaned reads were aligned to the human reference genome (GRCh38) using HISAT2 v2.1.0. Transcript assembly and gene quantification were performed with StringTie v2.1.3b, and differential expression analysis was conducted using DESeq2 in R. Differentially expressed genes were identified from the comparisons of MG vs. Control and MG+NAC vs. MG using an adjusted *P* value < 0.05 and an absolute log2 fold change > 0.75.

### RNA isolation and quantitative real-time PCR

Total RNA was isolated from *C. elegans* using the Direct-zol RNA MiniPrep Kit (Zymo Research, R2025) and from human cell lines and mouse brain tissues using the RNeasy Plus Mini Kit (Qiagen). cDNA was synthesized using the SuperScript IV Reverse Transcriptase (Invitrogen, 18090010) for *C. elegans* or avian myeloblastosis virus reverse transcriptase (New England Biolabs, M0277L) with oligo(dT)15 primer (Promega, C1101) for human and mouse samples. Quantitative real-time PCR (qPCR) was performed using TB Green Premix Ex Taq (Takara Bio, RR420A) on Rotor-Gene Q (Qiagen) or Thermal Cycler Dice (Takara Bio) systems. *rpl-35*, *ACTB*, and *Tbp* were used as internal reference genes for *C. elegans*, human, and mouse samples, respectively. Relative mRNA expression levels were calculated using the 2^−ΔΔCt^ method. Primer sequences are listed in Table S2.

### Mouse strains and growth conditions

A total of 18 wild-type male C57BL/6J mice (>48 weeks of age and weighing approximately 30 g) were purchased from Daehan Biolink (Eumseong, Republic of Korea). Mice were assigned to either the WT *E. coli*-colonized group (*n* = 8) or the Δ*hns E. coli*-colonized group (*n* = 10). The individual mouse was the experimental unit. Mice were housed under specific pathogen-free conditions at a density of 4–5 mice per cage, under a 12-h light/dark cycle, with ad libitum access to food and water.

Animals were randomly assigned to experimental groups. Blinding was not feasible during *E. coli* administration; subsequent experiments and analyses were performed by a different investigator blinded to group allocation. No unexpected adverse events were observed during the study. All animal experiments were approved by the Institutional Animal Care and Use Committee of Ajou University School of Medicine (IACUC No. 2024-0040) and conducted in accordance with institutional guidelines. This study was conducted and reported in accordance with the ARRIVE 2.0 guidelines.

### *E. coli* suspension preparation and gavage

WT *E. coli* and Δ*hns E. coli* were grown fresh for each feeding session and collected immediately prior to dosing. *E. coli* were resuspended in PBS and measured by absorbance at 600 nm to confirm the administered colony-forming units (CFUs). For intestinal microbiota depletion, prior to oral gavage with *E. coli*, mice were treated with 200 μL of an antibiotic cocktail containing vancomycin (100 mg/kg; Sigma-Aldrich, SBR00001), ampicillin (200 mg/kg), metronidazole (200 mg/kg; Alfa Aesar, J61499.14), and neomycin (200 mg/kg; Alfa Aesar, H60258.14) by oral gavage once daily for 7 days. Then, mice were administered 100 μL of *E. coli* suspension by oral gavage once a day for 14 days, with a total bacterial load of 10^8^ CFUs. Reusable stainless steel 20 G feeding needles (Jdbio, JD-S-124-70-20G) were used. Before and between gavages, the feeding needle was washed with filtered 70% ethanol and rinsed with sterile PBS. One group of feeding needles per treatment was used to avoid cross-contamination. Confirmation of bacterial dosing was conducted by collecting fecal pellets and resuspending them in PBS and then plating them on LB agar plates containing kanamycin (50 μg/mL; MB Cell, MB-K4390). Colonies were picked and genotyped.

### Mouse brain tissue preparation

For mRNA preparation, mice were sacrificed by cervical dislocation. The brains were quickly dissected and the striatum and SN were isolated on ice using an Alto mouse brain slicer matrix (Roboz Surgical Instruments, Gaithersburg, MD) and a micro-knife. The dissected regions were then put on dry ice and stored at −80 °C until use.

### Statistics

Statistical significance was assessed using methods appropriate to the experimental design. The chi-square (χ^2^) test was used to compare severity category distributions in *C. elegans* assays, and lifespan data were analyzed using the log-rank (Mantel–Cox) test. The Mann–Whitney U test was used for group comparisons in fecal microbiota analyses. For Figure 1, planned pairwise comparisons were performed using two-sided Mann–Whitney U tests with Bonferroni correction within each gene, and ordered trends across groups were assessed using the Jonckheere–Terpstra trend test with FDR-adjusted q values and Kendall’s tau-b effect sizes. Plasma MG levels between the body-first and brain-first groups were compared using a two-tailed Welch’s t-test, whereas all other pairwise comparisons were analyzed using two-tailed Student’s t-tests. Associations between continuous variables were assessed using Pearson correlation analysis. Pearson’s r is presented for unadjusted correlations, except in Figure 1C, where r^2^ is shown. For analyses of plasma MG levels in relation to cardiac MIBG uptake, partial Pearson correlation coefficients adjusted for diabetes mellitus, age, and sex were calculated using the ppcor package in R, and 95% confidence intervals were derived from Fisher’s z transformation. Multivariable linear regression was performed as a sensitivity analysis. Clinical characteristics (Table S1) were compared between body-first and brain-first PD using the Mann–Whitney U test for continuous variables and the chi-square or Fisher’s exact test for categorical variables. Differences in correlation coefficients between the body-first and brain-first groups were assessed using Fisher’s r-to-z test and multivariable linear regression including an MG-by-subtype interaction term, adjusted for diabetes mellitus, age, and sex. Sensitivity analyses included partial Spearman correlations, log-transformed MG, and exclusion of high-MG observations above Q3 + 1.5 × IQR within each subtype. The Benjamini–Hochberg procedure was applied across the six subtype-by-MIBG comparisons.

## Results

### Increased gut microbial *hns* in the stool of body-first PD patients

Given the emerging role of microbially derived MG in host physiology,^15^ ^,^ ^19^ we first investigated its clinical relevance to PD. To this end, we profiled the fecal microbiomes of 36 patients with PD (15 body-first, 9 brain-first, and 12 undetermined) and 36 age- and sex-matched healthy controls from a well-characterized Korean cohort (hereafter, the metagenomic cohort), whose detailed clinical phenotypes have been described previously.^7^ Strikingly, *hns*, a key microbial regulator of MG production,^18^ was significantly elevated in PD patients, whereas it was present at very low abundance in healthy controls (detected in 31 of 36; median 0.005 RPKM) (Figure 1A). Notably, *hns* abundance was most prominent in the body-first PD subgroup, classified based on the presence of premotor RBD and reduced cardiac MIBG uptake.^3^ To assess whether this pattern depended on the operational definition, we repeated the *hns* analysis using premotor RBD alone and cardiac MIBG uptake alone. Under either single-marker definition, both resulting PD groups showed higher *hns* abundance than healthy controls after Bonferroni correction (all adjusted *P* < 0.001), with numerically higher abundance in the groups classified as body-first by the respective marker—that is, premotor RBD or reduced cardiac MIBG uptake (Figure S1). Thus, the overall PD-associated enrichment of *hns* was maintained across alternative definitions, whereas subtype separation was most pronounced under the concordant dual-marker definition.

To identify the microbial origin of elevated *hns*, we investigated which bacterial taxa contributed to the abundance of *hns* in the feces of PD patients. Among the gut microbiota, *Escherichia* species were identified as the predominant carriers of *hns* genes in PD patients, representing 32% of *hns*-encoding bacteria (Figure 1B), a pattern mirrored by the significant positive correlation observed between *hns* levels and *E. coli* abundance (Figure 1C). These observations suggest that the low *hns* signal in controls is consistent with the low abundance of *hns*-carrying taxa, particularly *Enterobacteriaceae* and *Escherichia*/*E. coli*, rather than the true absence of *hns* itself.

In addition to *hns*, notable changes in MG metabolic pathways (Figure 1D) were observed in PD patients, particularly in the body-first subtype. The MG-producing genes *mgsA* and *tpiA* were significantly upregulated (Figure 1E and F), while key upstream detoxification genes *gloA* and *ydjG* ^25^ ^,^ ^26^ showed a significant decreasing trend across the ordered groups (healthy controls to body-first PD; Jonckheere–Terpstra test), although the body-first versus healthy-control difference did not survive Bonferroni correction (Figure 1G and H). These changes, alongside increased *hns* abundance, are consistent with a metagenomic signature of increased MG-producing potential rather than direct measurement of intestinal MG burden. In contrast, several downstream detoxification genes, including *yeiG*, *dld*, *aldA*, and *lldD*, ^27-29^ were significantly upregulated, likely reflecting a compensatory response to reduced upstream activity (Figure 1I–L). *gloB/C* showed a weak but statistically significant increasing trend (Figure 1M), and *hchA* showed a similar trend (Figure 1N).

Collectively, these findings indicate that *hns* and other microbial genes associated with MG metabolism are altered in the feces of PD patients, particularly those with the body-first subtype, in a manner consistent with increased MG-producing potential rather than direct measurement of intestinal MG levels, and these microbial shifts become progressively more pronounced from healthy controls through brain-first and undetermined to body-first PD (Jonckheere–Terpstra trend).

### Higher plasma MG in body-first than in brain-first PD

To investigate whether the altered profile of fecal MG-regulating genes, including *hns*, is linked to host MG changes, we analyzed plasma MG levels in a separate Korean PD cohort (hereafter, the plasma MG cohort), distinct from the metagenomic cohort described above and comprising 81 patients with PD (46 body-first, 16 brain-first, and 19 undetermined). This cohort was used exclusively for plasma MG measurements, and no fecal microbiome analysis was performed in these patients. Detailed demographic and clinical characteristics, including age, sex, disease duration, motor severity, constipation, and probiotic use, are provided in Table S1. Parallel to fecal microbial gene shifts, an inter-subtype analysis of PD patients revealed that plasma MG levels were significantly higher in the body-first group than in the brain-first group (Figure 2A).

**Figure 2.**
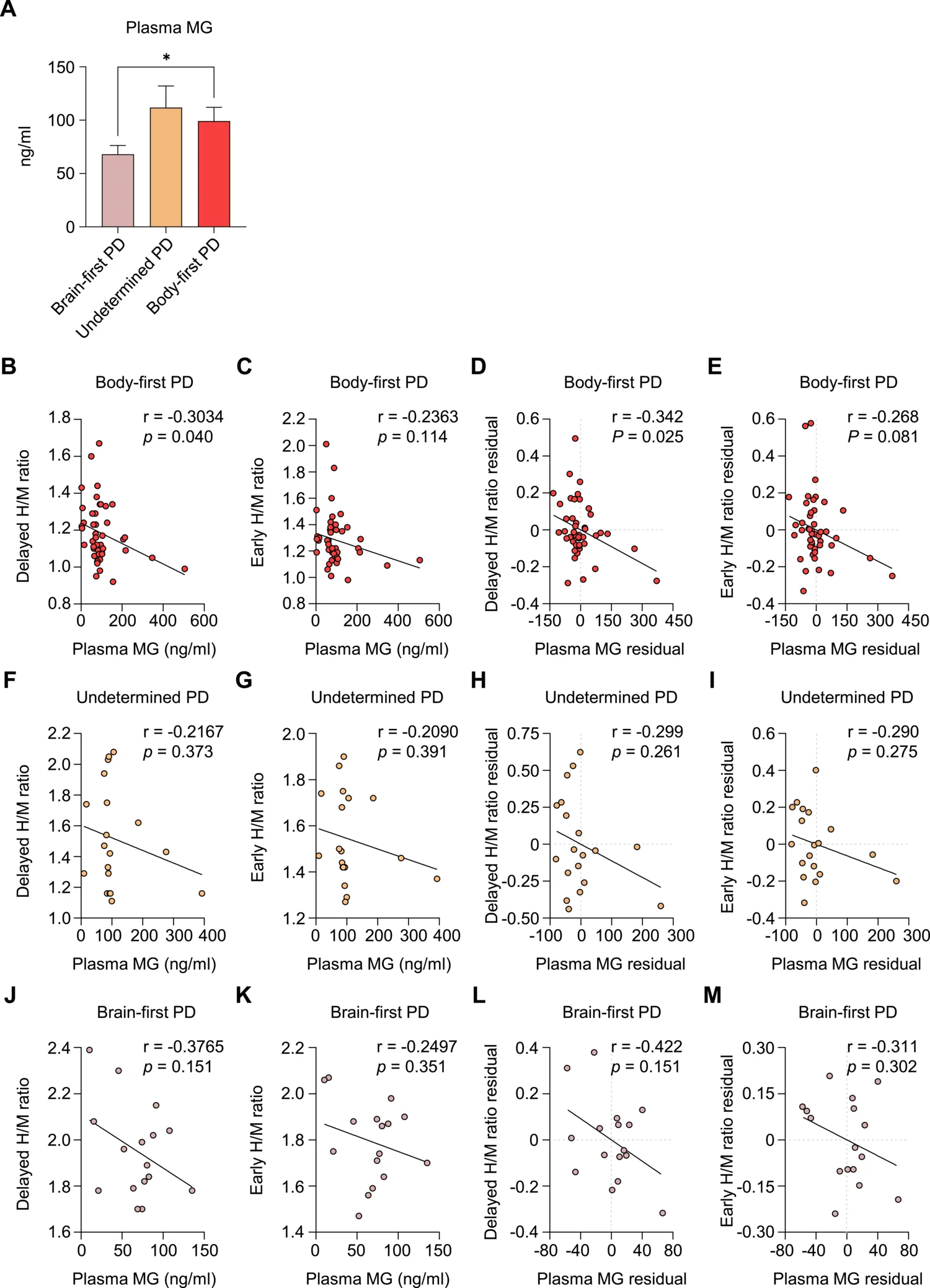
Higher plasma MG in body-first than in brain-first PD. (A) Plasma MG levels across PD subtypes. Plasma MG levels were significantly higher in the body-first PD group compared to the brain-first PD group. Data are presented as mean ± SE. Statistical significance was determined using Welch’s t-test for group comparisons. *P < 0.05 (plasma MG cohort; *n* = 16 for brain-first PD, *n* = 19 for undetermined PD, and *n* = 46 for body-first PD). (B–E) Correlation analyses between plasma MG levels and cardiac MIBG uptake in body-first PD patients. Plasma MG levels were inversely correlated with the delayed H/M ratio in both unadjusted (r = −0.3034, *P* = 0.040) (B) and adjusted analyses (partial r = −0.342, *P* = 0.025) (D), whereas no significant correlation was observed with the early H/M ratio in either unadjusted (r = −0.2363, *P* = 0.114) (C) or adjusted analyses (partial r = −0.268, *P* = 0.081) (E). Multiplicity and sensitivity analyses are provided in Supplementary Table S3. (F–I) Correlation analyses between plasma MG levels and cardiac MIBG uptake in undetermined PD patients. No significant correlation was observed between plasma MG levels and either delayed or early H/M ratios in unadjusted (F, G; delayed: r = −0.2167, *P* = 0.373; early: r = −0.2090, *P* = 0.391) or adjusted analyses (H, I; delayed: partial r = −0.299, *P* = 0.261; early: partial r = −0.290, *P* = 0.275). (J–M) Correlation analyses between plasma MG levels and cardiac MIBG uptake in brain-first PD patients. No significant correlation was observed between plasma MG levels and either delayed or early H/M ratios in unadjusted (J, K; delayed: r = −0.3765, *P* = 0.151; early: r = −0.2497, *P* = 0.351) or adjusted analyses (L, M; delayed: partial r = −0.422, *P* = 0.151; early: partial r = −0.311, *P* = 0.302).

To further investigate the clinical significance of higher plasma MG in body-first PD, we examined the relationship between plasma MG levels and delayed cardiac MIBG H/M ratio (delayed H/M ratio), a quantitative marker of cardiac sympathetic denervation in body-first PD.^3^ Within body-first PD, plasma MG was inversely correlated with the delayed H/M ratio in the unadjusted analysis (r = −0.303, nominal *P* = 0.040; Figure 2B) and after adjustment for diabetes mellitus, age, and sex (partial r = −0.342, nominal *P* = 0.025; 95% CI, −0.58 to −0.05; Figure 2D). The MG-by-subtype interaction was not significant (*P* = 0.202). The direction of the association remained inverse in analyses using log-transformed MG, rank-based correlations, and exclusion of high-MG observations, although its magnitude and statistical significance were attenuated. The association did not remain significant after Benjamini–Hochberg correction across the six subtype-by-MIBG comparisons (BH-adjusted *P* = 0.148; Table S3).

### Deletion of *hns* attenuates host dopaminergic neurodegeneration

Given that gut microbial *hns* exhibited the most prominent clinical contrast in our patient cohort, being present at only very low abundance in healthy controls but highly enriched in body-first PD (Figure 1A), we next examined whether its absence could alleviate host dopaminergic neurodegeneration. To test the functional impact of this PD-associated bacterial gene signature, we utilized a *C. elegans in vivo* model fed with Δ*hns E. coli* to deplete microbial *hns* activity. Direct measurement confirmed that Δ*hns E. coli* produced significantly less MG than WT *E. coli* (Figure 3A and B), supporting *hns*-dependent regulation of bacterial MG production under our experimental conditions. *C. elegans* was selected because of its well-characterized dopaminergic nervous system (consisting of four CEP and two ADE head neurons) (Figure 3C) and its established utility in studying host–microbiota interactions relevant to neurodegeneration.^30-32^ Two widely used *C. elegans* PD models were employed: the αSyn model, genetically engineered to express human αSyn in dopaminergic neurons (Figure 3C), and the 6-hydroxydopamine (6-OHDA) model, chemically induced by 6-OHDA (Figure 3F).^33-38^ Both models express GFP in dopaminergic neurons, allowing visualization of neuronal integrity.

**Figure 3.**
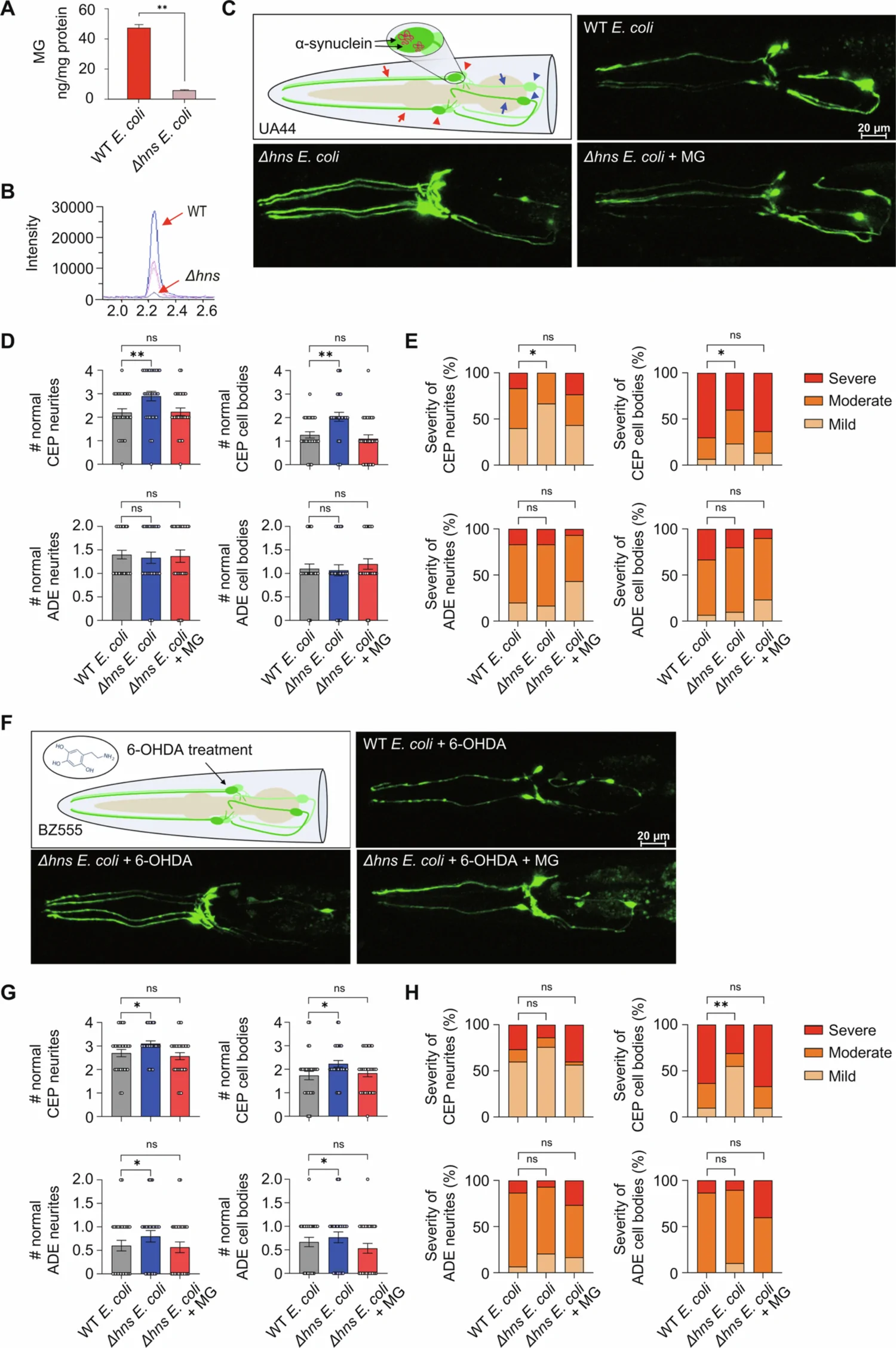
Deletion of *hns* attenuates host dopaminergic neurodegeneration. (A) MG levels in WT and Δ*hns E. coli*. MG levels were significantly lower in Δ*hns E. coli* than in WT *E. coli*. (B) Representative LC-MS/MS chromatograms of MG-derived 2-MQ in WT and Δ*hns E. coli* lysates. Blue, analyte (2-MQ); pink, internal standard (IS, ^13^C₃-2-MQ). Data are presented as mean ± SE. Statistical significance was determined using Student’s t-test. *P* < 0.01. *n* = 3 per group. (C-E) αSyn-overexpressing dopaminergic neuron model (αSyn model) using UA44 [*dat-1p*::αSyn; *dat-1p*::GFP]. Representative fluorescence images of dopaminergic neurons in the αSyn model. The top left illustration depicts the experimental design using the UA44 strain expressing αSyn and GFP in dopaminergic neurons. Red arrows indicate CEP neurites, and red arrowheads indicate CEP cell bodies. Blue arrows indicate ADE neurites, and blue arrowheads indicate ADE cell bodies. Representative images show dopaminergic neurons in worms fed with WT *E. coli*, Δ*hns E. coli*, or Δ*hns E. coli* supplemented with 10 mM MG (C). Quantification of intact CEP and ADE neurites and cell bodies in worms under each condition (D). Proportion of worms exhibiting mild, moderate, or severe neuronal damage based on morphological assessment (E). (F-H) 6-OHDA-induced dopaminergic neuron degeneration model (6-OHDA model) using BZ555 [*dat-1p*::GFP]. Representative fluorescence images of dopaminergic neurons in the 6-OHDA model. The top left illustration depicts the experimental design using the BZ555 strain expressing GFP in dopaminergic neurons. Representative images show dopaminergic neurons in worms fed with WT *E. coli*, Δ*hns E. coli*, or Δ*hns E. coli* supplemented with 10 mM MG (F). Quantification of intact CEP and ADE neurites and cell bodies in worms under each condition (G). Proportion of worms exhibiting mild, moderate, or severe neuronal damage based on morphological assessment (H). All quantifications were performed using 30 worms per group (*n* = 30). Data are presented as mean ± SE. Statistical significance was determined using Student’s *t*-test for group comparisons and chi-square test for proportion analysis. *P < 0.05, **P < 0.01.

In the αSyn model, feeding with Δ*hns E. coli* significantly increased the number of intact CEP neurites and cell bodies compared to worms fed with WT *E. coli* (Figure 3D). Consistent with its previously reported strong impact on MG reduction,^18^ Δ*hns E. coli* exhibited a marked neuroprotective effect among other elevated MG metabolism-related *E. coli* mutants in our PD cohort (including Δ*mgsA,* Δ*dld,* Δ*lldD, and* Δ*aldA*) (Figure S2A–D). The proportion of worms exhibiting dopaminergic neuron damage was also significantly reduced in the Δ*hns* group, particularly in CEP neurites and cell bodies (Figure 3E). Importantly, exogenous MG supplementation abolished the protective effects of Δ*hns E. coli*, resulting in fewer intact dopaminergic neurites and increased neuronal degeneration (Figure 3D and E). We next examined these findings in the 6-OHDA model. Similar to the αSyn model, feeding with Δ*hns E. coli* significantly increased intact CEP neurites and cell bodies and reduced dopaminergic neuron damage compared with WT *E. coli* (Figure 3G and H). MG supplementation again reversed the neuroprotective effects of Δ*hns E. coli*. Across both PD models, ADE neurons exhibited a less pronounced trend (Figure 3D, E, G, and H).

To evaluate whether these effects extended beyond protection of dopaminergic neurons, we assessed lifespan and locomotor behavior in both PD models. Δ*hns E. coli* extended lifespan and improved thrashing behavior compared to WT *E. coli*, whereas MG treatment suppressed these benefits (Figure S3A–E). These findings suggest that deletion of MG-producing regulator *hns* not only protects against dopaminergic neurodegeneration but also improves overall survival and motility in *C. elegans* PD models.

### *rig-5* mediates the neuroprotective effects of *hns* deletion

Next, we sought to identify the host genes responsible for rescuing dopaminergic neuron degeneration in response to the deletion of microbial *hns*. To achieve this, we reanalyzed bulk RNA sequencing data from WT *C. elegans* fed with Δ*hns* mutant *E. coli*, which had been included in a previous study.^18^ We identified 695 genes with significantly altered expression in worms fed with Δ*hns E. coli*. To gain further insight into the biological functions associated with these genes, we performed gene set enrichment analysis (GSEA). GSEA highlighted the enrichment of categories related to “neuron projection” and “synapse” (Figure S4A). We then examined the genes contributing to these gene ontology (GO) and found that eight genes associated with “neuron projection,” four with “synapse,” and four overlapping both categories were significantly upregulated in the Δ*hns* group (Figure S4B–D). To further investigate whether these 16 genes are also upregulated in PD models of *C. elegans*, we examined their mRNA levels. Interestingly, the gene *rig-5*, encoding an immunoglobulin-like domain-containing protein involved in axon navigation,^39^ was the most highly upregulated gene among those examined in both PD models, with a statistically significant increase in expression (Figure 4A and B). To determine whether *rig-5* plays a critical role in the dopaminergic neuron rescue effect mediated by Δ*hns* mutant *E. coli*, we suppressed the expression of *rig-5* and assessed dopaminergic neuron integrity. Strikingly, *rig-5* knockdown abolished the neuroprotective effect of Δ*hns E. coli* on CEP neurites and cell bodies in both PD models (Figure 4C–H). Consistent with its role in neuronal protection, *rig-5* was also required for the improvement in locomotor behavior but not for the lifespan extension induced by Δ*hns* mutant *E. coli* (Figure S3F–J). These findings suggest that *rig-5* is a key gene mediating the neuroprotective and behavioral benefits induced by microbial *hns* deletion.

**Figure 4.**
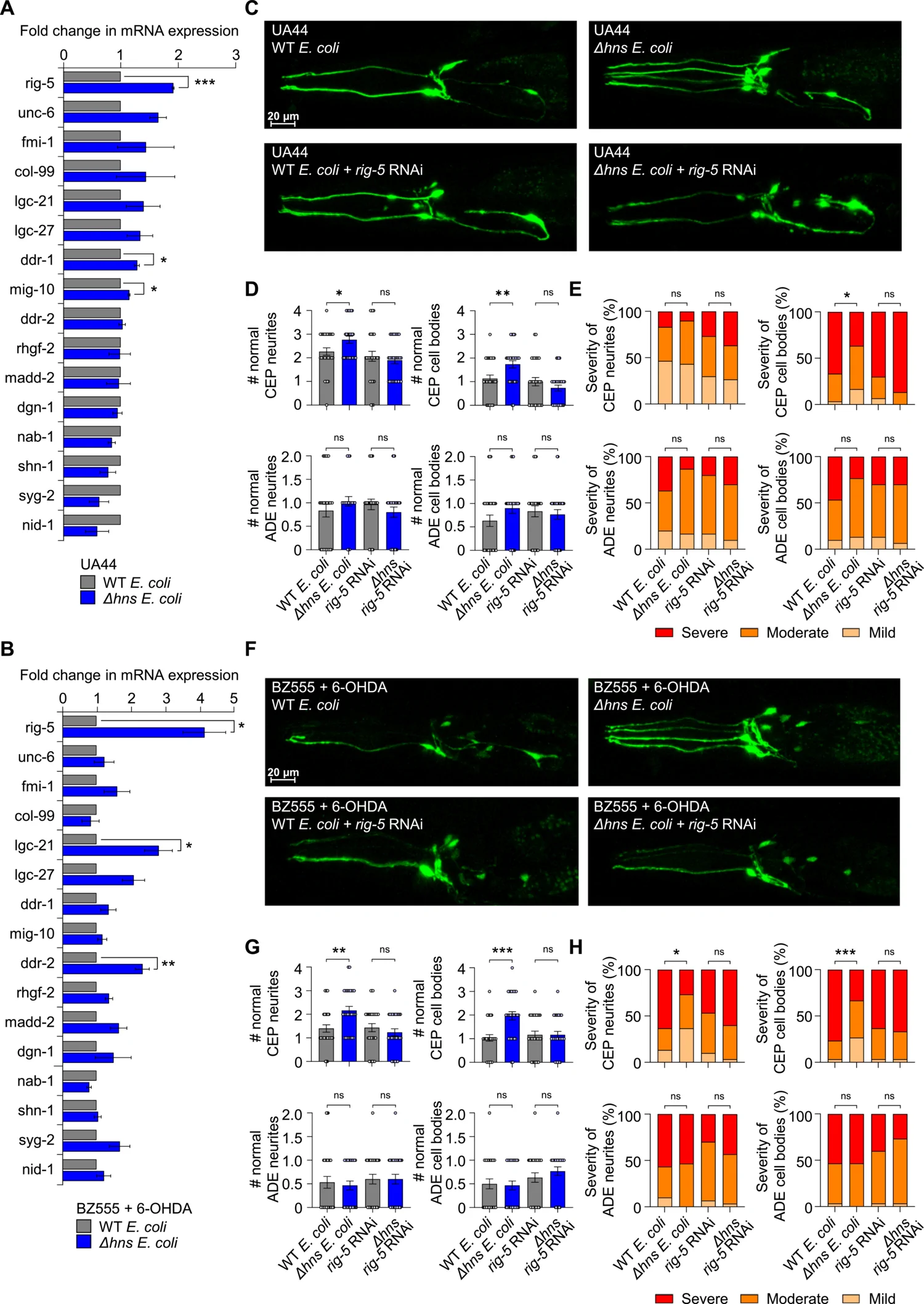
*rig-5* mediates the neuroprotective effects of *hns* deletion. (A, B) Fold change in mRNA expression of genes associated with the GO terms “synapse” and “neuron projection” in *C. elegans* PD models. In both the αSyn model (A) and the 6-OHDA model (B), *rig-5* was the most highly upregulated gene in worms fed with Δ*hns E. coli*. (C) Representative fluorescence images of dopaminergic neurons in αSyn model fed with WT *E. coli*, Δ*hns E. coli*, *rig-5* RNAi-expressing WT *E. coli*, or *rig-5* RNAi-expressing Δ*hns E. coli* . (D-E) Quantification of intact CEP and ADE neurites and cell bodies (D) and proportion of worms exhibiting mild, moderate, or severe neuronal damage (E). (F) Representative fluorescence images of dopaminergic neurons in 6-OHDA model fed with WT *E. coli*, Δ*hns E. coli*, *rig-5* RNAi-expressing WT *E. coli*, or *rig-5* RNAi-expressing Δ*hns E. coli* . (G-H) Quantification of intact CEP and ADE neurites and cell bodies (G) and proportion of worms exhibiting mild, moderate, or severe neuronal damage (H). All quantifications were performed using 30 worms per group (*n* = 30). Data are presented as mean ± SE. Statistical significance was determined using Student’s *t*-test for group comparisons and chi-square test for proportion analysis. *P < 0.05, **P < 0.01, ***P < 0.001.

### MG-dependent regulation of *rig-5* orthologs in SH-SY5Y human neuroblastoma cells

To explore potential human counterparts of *rig-5*, we identified nine putative mammalian orthologs—*IGLON5*, *IGSF5*, *IGSF9B*, *LSAMP*, *NEGR1*, *NTM*, *OPCML*, *ROBO1*, and *TMIGD1*—using the HGNC Comparison of Orthology Predictions (HCOP) tool (Figure S5A).^40^ We then assessed the transcriptional changes of these orthologs in SH-SY5Y human neuroblastoma cells (SH-SY5Y cells), which exhibit dopaminergic neuronal characteristics,^41^ following direct treatment with MG. Bulk RNA-sequencing analysis (RNA-seq) revealed that *LSAMP* was significantly downregulated by MG, and this transcriptomic alteration was reversed by *N*-acetylcysteine (NAC), an antioxidant and glutathione precursor that can facilitate cellular MG detoxification, supporting the involvement of MG-associated oxidative stress (Figure 5A). RT-qPCR validation confirmed these findings and further showed that the observed changes were dose dependent (Figure 5B). Although NAC-mediated recovery of *NEGR1* did not reach statistical significance in the initial RNA-seq (Figure 5A), RT-qPCR confirmed MG-induced suppression and NAC-mediated restoration of *NEGR1* expression (Figure 5B). In contrast, RNA-seq-identified changes in *IGSF5* were not validated (Figure S5B).

**Figure 5.**
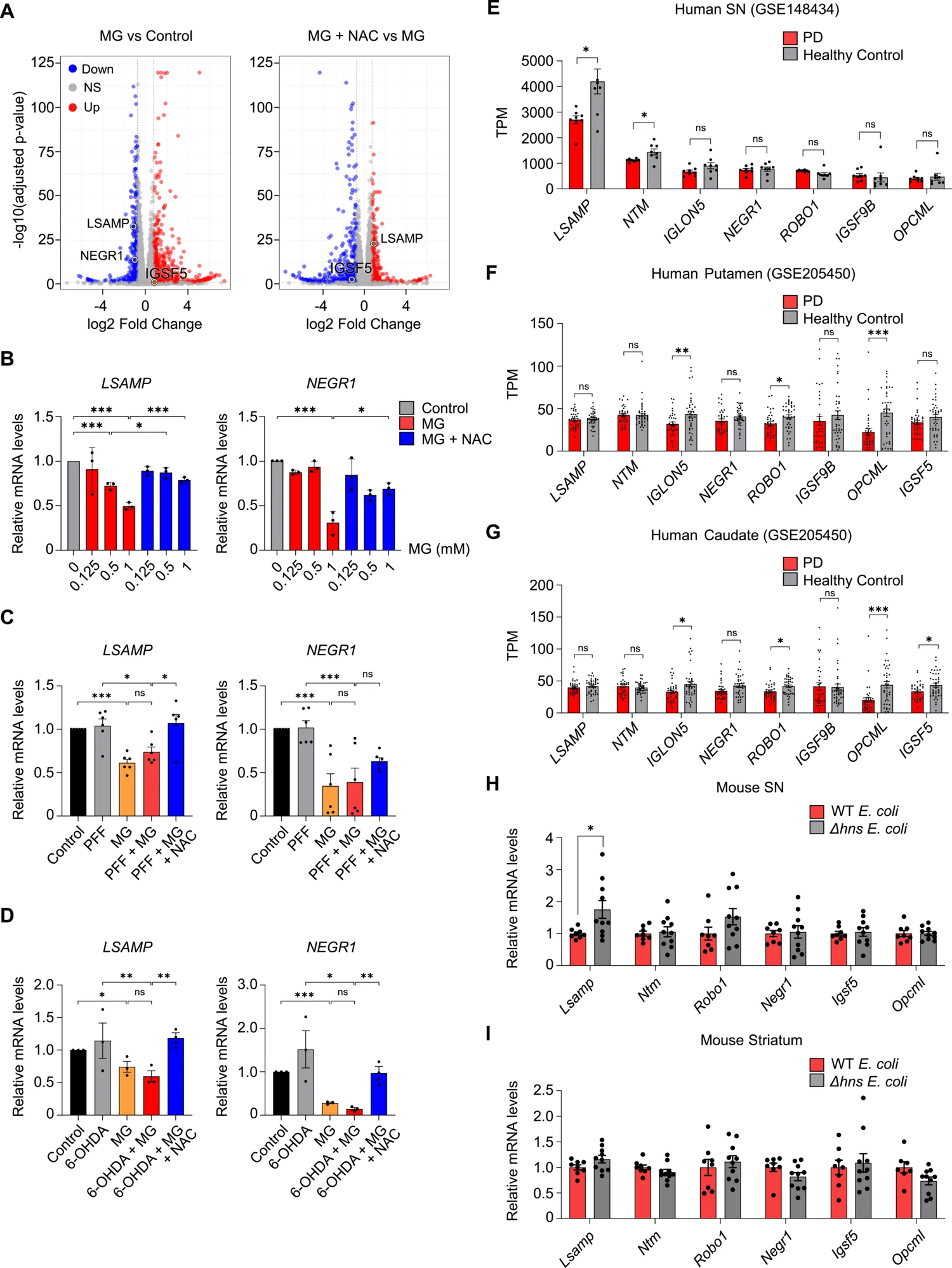
Investigation of *rig-5* orthologs in mammalian systems. (A) Differential gene expression analysis of SH-SY5Y cells treated with 1 mM MG for 24 h, with or without 10 mM NAC. Volcano plots display differentially expressed genes (DEGs) for MG vs. Control (left) and MG+NAC vs. MG (right), with upregulated (Up), downregulated (Down), and non-significant (NS) genes indicated in red, blue, and gray, respectively. Among the human orthologs of *C. elegans rig-5*, the significantly altered genes (*LSAMP*, *NEGR1*, and *IGSF5*) are highlighted with thick white outlines. (B) Relative mRNA levels of *LSAMP* (left) and *NEGR1* (right) in SH-SY5Y cells treated with various concentrations of MG (0, 0.125, 0.5, and 1 mM) for 24 h, with or without 10 mM NAC. (C, D) Relative mRNA levels of *LSAMP* and *NEGR1* in SH-SY5Y cells treated with 250 nM αSyn PFFs (C) or 100 *μ*M 6-OHDA (D) for 24 h, with or without 1 mM MG or 10 mM NAC. (E–G) Expression levels of *rig-5* orthologs in human brain regions, including the SN (E), putamen (F) and caudate (G), in PD patients and healthy controls. (H, I) Relative mRNA levels of *rig-5* orthologs in the SN (H) and striatum (I) of WT mice (>48 weeks of age) colonized with either WT or Δ*hns E. coli.* Data are presented as mean ± SE. Statistical significance was determined using Student’s *t*-test for group comparisons. *P < 0.05, **P < 0.01, ***P < 0.001.

We further examined the effects of MG on the two validated *rig-5* orthologs, *LSAMP* and *NEGR1*, under PD-like conditions induced by αSyn preformed fibrils (PFFs) or 6-OHDA. Neither αSyn PFFs nor 6-OHDA alone altered *LSAMP* or *NEGR1* expression, nor did they enhance the effects of MG (Figure 5C and D). Together, these results indicate that MG is sufficient to downregulate *LSAMP* and *NEGR1* expression, and this effect is not further enhanced by PD-relevant insults.

### Expression analysis of *rig-5* orthologs in human PD brains

Next, we analyzed bulk RNA-seq data from PD patients and healthy controls. In the SN dataset (GSE148434),^42^ *LSAMP* was the most highly expressed ortholog in healthy controls, and its expression was significantly reduced in PD patients. Among the remaining orthologs, most did not show significant changes, although *NTM* exhibited a modest but statistically significant reduction (Figure 5E). Orthologs with low expression levels, such as *IGSF5* and *TMIGD1*, were excluded from the datasets due to low or undetectable TPM values. In the striatum, several orthologs, including *IGLON5*, *ROBO1*, and *OPCML*, were significantly reduced in both the putamen and caudate, whereas *LSAMP* and *NTM* remained unchanged in both regions (GSE205450; Figure 5F and G).^43^ Together, these findings indicate that multiple *rig-5* orthologs may be altered in a region-dependent manner in PD.

### Modulation of *Lsamp* expression by gut microbial *hns* in mouse brain

Given that MG alone, rather than PD-relevant insults, downregulated *LSAMP* expression (Figure 5B−D), the reduced *LSAMP* expression observed in the SN of PD patients (Figure 5E) may be linked to the microbial *hns*–MG pathway. To assess this possibility, we investigated whether gut microbial *hns* modulates *rig-5* ortholog expression *in vivo* using a mouse *E. coli* colonization model. Following colonization of antibiotic-pretreated WT C57BL/6 mice (>48 weeks of age) with either WT or Δ*hns E. coli*, RT-qPCR analysis revealed that *Lsamp* was the only ortholog affected among those tested. In an exploratory analysis of available residual plasma samples from this cohort, plasma MG was lower in the Δ*hns E. coli* group than in the WT *E. coli* group (Figure S6), providing supportive supplementary evidence consistent with the bacterial *hns*–MG link. Specifically, its expression was significantly increased in the SN of mice colonized with *∆hns E. coli* (Figure 5H), while no differences were observed in the striatum (Figure 5I). This regional pattern of *Lsamp* expression in *E. coli*-colonized mice was consistent with that observed in PD patients (Figure 5E−G). Other orthologs, including *Ntm, Robo1, Negr1, Igsf5*, and *Opcml,* did not exhibit significant changes. Orthologs with undetectable expression were excluded from the analysis. Collectively, our findings support *in vivo* regulation of a candidate mammalian host gene node by bacterial *hns*-dependent activity.

## Discussion

Despite growing evidence linking the gut microbiota to PD,^9^ ^,^ ^10^ ^,^ ^44-50^ few studies have identified a defined microbial gene that mechanistically links bacterial metabolism to host neuronal responses. Here, we identify a gut microbial *E. coli hns*–MG pathway linking *rig-5* regulation to dopaminergic vulnerability in *C. elegans*, with corresponding modulation of its candidate mammalian ortholog, *LSAMP*. This conclusion is supported by evidence from an integrative, cross-species experimental framework encompassing PD subtype-specific metagenomics, plasma MG measurements, *C. elegans* PD models, analyses of PD patient brains, human neuroblastoma cells, and mouse colonization studies.

Building on our previous metagenomic analysis of PD subtypes,^7^ the current study newly highlights *hns* as a microbial gene elevated in the stool of PD patients, particularly those with the body-first subtype (Figure 1A). Consistent with this observation, several MG-producing genes were increased, whereas upstream MG-detoxification genes showed decreasing trends (Figure 1E−H), supporting a microbial MG-related gene profile consistent with increased MG-producing potential. Although PD subtype definitions remain heterogeneous, we adopted an a priori concordant framework combining premotor RBD and abnormal cardiac MIBG uptake, which showed the highest predictive accuracy in our previous study.^7^ Discordant cases were classified as undetermined to reduce misclassification and preserve phenotypic contrast. Single-marker sensitivity analyses confirmed overall *hns* enrichment but showed weaker subtype separation (Figure S1), supporting the utility of the dual-marker framework. Within this pre-specified framework, the pronounced enrichment of *hns* in body-first PD supports the prediction that the *hns*–MG pathway may be more relevant to this subtype than to PD more broadly. However, this classification may have reduced sensitivity to intermediate phenotypes, and potential involvement of this pathway in brain-first PD cannot be excluded. Prospective subtype-stratified and prodromal studies are therefore warranted to define the temporal and subtype-specific relevance of this pathway and to determine whether its modulation could enable early, personalized microbiome-targeted intervention.

In a separate plasma MG cohort, MG levels were higher in body-first than in brain-first PD (Figure 2A), although the lack of healthy controls precluded conclusions about an absolute increase in PD. Within body-first PD, plasma MG showed a nominal inverse association with delayed cardiac MIBG uptake (Figure 2B and D); however, this association did not differ significantly between subtypes and was weakened in sensitivity and multiple-comparison analyses. Interpretation of the subtype comparison was further limited by the smaller brain-first group. MG levels were also relatively high in the undetermined group; notably, 13 of 19 patients had abnormal cardiac MIBG uptake without premotor RBD, with descriptively higher and more variable MG levels than those with premotor RBD but normal MIBG uptake. This pattern suggests that systemic MG may be more closely related to peripheral autonomic involvement than to categorical subtype assignment. Overall, these findings remain supportive but exploratory and require confirmation in larger, subtype-balanced cohorts with paired microbiome–plasma measurements and healthy controls.

Although MG has primarily been studied as a host-derived metabolite in PD,^11^ ^,^ ^15^ ^,^ ^16^ ^,^ ^51^ our findings support bacterial *hns*-dependent MG production and its functional relevance to dopaminergic neurodegeneration in *C. elegans*. Feeding *C. elegans* with Δ*hns E. coli*, which produces reduced levels of MG (Figure 3A and B), protected dopaminergic neurons in both αSyn and 6-OHDA models (Figure 3C−H), with a more robust effect in CEP than in ADE neurons. This preferential protection of CEP neurons may reflect subtype-specific differences in anatomical position, exposure to bacterial or metabolic cues, and potential intrinsic susceptibility to MG-related stress, whereas the weaker effects in ADE neurons warrant cautious interpretation and further mechanistic investigation. Exogenous MG abolished the protection conferred by Δ*hns E. coli*, implicating MG in *hns*-dependent bacterial toxicity, while not excluding the involvement of other H-NS-regulated pathways. Exploratory analysis of residual plasma from the mouse colonization cohort showed lower plasma MG in the Δ*hns E. coli* group (Figure S6). This finding is consistent with an association between bacterial MG-producing capacity and *in vivo* MG alterations, but it does not establish that the circulating MG difference originated directly from the administered bacterial strain. The need for source-resolved MG tracing is discussed further in the Limitations.

Mechanistically, we identified *rig-5* as a key host effector of the *hns*–MG pathway in *C. elegans*. Exposure to Δ*hns E. coli* upregulated *rig-5*, and *rig-5* knockdown abolished Δ*hns*-mediated neuroprotection (Figure 4). Cross-species analyses identified putative mammalian counterparts of this response. In SH-SY5Y cells, MG suppressed the candidate *rig-5* orthologs *LSAMP* and *NEGR1* (Figure 5A and B). In the PD brain, multiple *rig-5* orthologs were altered, with *LSAMP* and *NTM* notably reduced in the SN (Figure 5E–G). Colonization with Δ*hns E. coli*, in contrast, increased *Lsamp* expression in the mouse SN (Figure 5H and I). Importantly, *LSAMP* showed the most consistent evidence across the mammalian systems examined, supporting its prioritization as a candidate mammalian host gene node, although the *rig-5*–*LSAMP* correspondence requires direct functional validation. The reduction of *NTM* in the PD SN also warrants further investigation. *Lsamp* also showed the highest basal expression in the SN among the brain regions examined (Figure S5C), suggesting possible regional sensitivity to microbial *hns*/MG-dependent regulation in this region. Together, these cross-species findings support a potential gut–brain pathway in which bacterial *hns* status and MG availability influence *rig-5*/*LSAMP* expression, providing a plausible link between microbial metabolism and SN-associated host gene regulation in PD.

*LSAMP*, *NTM*, and *NEGR1* belong to the IgLON family of cell adhesion molecules involved in neuronal connectivity and synaptic organization.^52-54^ Although their roles in PD remain poorly understood, previous studies have linked these molecules to αSyn-related processes, including correlations between NEGR1 and cerebrospinal fluid αSyn levels^55^ and the identification of LSAMP and NTM as potential αSyn-binding partners.^56^ Our findings extend these observations by identifying *LSAMP* as a mammalian host gene node responsive to MG-associated stress and bacterial *hns*-dependent regulation.

These findings also suggest several translational directions. Recent advances in phage-delivered *in situ* editing of specific gut bacterial genes, exemplified by targeting the curli gene *csgA*,^57^ raise the possibility, although still conceptual and requiring careful validation, of selectively modulating *hns*+ *E. coli* or *hns*-linked downstream MG-producing pathways to reduce microbial MG production without broadly disrupting the gut microbiota. However, because H-NS regulates a broad bacterial gene network,^58^ more precise intervention may be achieved by identifying downstream MG-producing or MG-detoxifying pathways influenced by *hns* or by directly targeting MG-related enzymes such as *mgsA*. Complementary approaches could include the development or repurposing of MG-lowering compounds and interventions that preserve or restore LSAMP expression or signaling. Metformin and pyridoxamine, for example, have been reported to lower circulating MG in humans, but neither acts selectively on MG, and their therapeutic CNS exposure and relevance to PD remain uncertain. Similarly, LSAMP-based therapies will require tractable delivery strategies and *in vivo* evidence of neuroprotective efficacy. Successful clinical translation will therefore depend on selective microbiome modulation without dysbiosis, effective reduction of MG in relevant gut, systemic, or CNS-associated compartments, and confirmation that restoring LSAMP-related signaling modifies PD-relevant neurodegenerative phenotypes.

## Limitations

Although *LSAMP* was identified as the most consistent candidate mammalian counterpart/ortholog of *rig-5*, its regulation by MG and its role in dopaminergic vulnerability in mammalian systems remain incompletely defined. Our NAC experiments implicate MG-associated oxidative stress in *LSAMP* suppression, but the underlying mechanisms and potential contributions of inflammatory or epigenetic pathways require further clarification. The present mouse colonization experiment was designed to assess whether bacterial *hns*-dependent activity can modulate candidate host gene expression in the mammalian SN, rather than to test PD-related dopaminergic neurodegeneration. Accordingly, the current mammalian *in vivo* data should be interpreted as evidence of *Lsamp* regulation, not as evidence of dopaminergic neuroprotection or disease modification in a mammalian PD model. Direct validation of the *hns*–MG–LSAMP pathway in a mammalian PD model therefore remains an important next step. Given the body-first framework of the present study, future work will prioritize a gut-seeded αSyn PFF model, in which αSyn pathology is initiated in the gastrointestinal tract and can propagate along the gut–brain axis.^59^ ^,^ ^60^ Combining this model with WT versus Δ*hns E. coli* colonization will enable assessment of whether bacterial *hns*-dependent activity influences αSyn pathology, nigrostriatal degeneration, *LSAMP/Lsamp* regulation, and PD-related motor or autonomic phenotypes. Because such experiments require long-term model establishment, bacterial colonization, pathological staging, behavioral assessment, and molecular validation, they could not be incorporated into the present study but represent a key direction for future work.

The interpretation of *in vivo* MG changes is also limited by the lack of source-resolved MG tracing. Although Δ*hns E. coli* produced lower MG than WT *E. coli* in bacterial lysates, and residual plasma samples from the mouse colonization cohort showed lower plasma MG in the Δ*hns E. coli* group, these data do not establish that the circulating MG difference originated directly from the administered bacterial strain. Thus, we interpret these findings as evidence that bacterial MG-producing capacity is associated with *in vivo* MG alterations, rather than as definitive proof of bacterial-source causality. Future studies should correlate strain-level colonization burden with fecal and plasma MG concentrations and incorporate stable-isotope tracing, pathway-specific MG metabolism inhibitors, or bacterial MG reporter strains to distinguish microbial from host-derived MG pools *in vivo*.

Fecal microbiota transplantation will also be important for evaluating whether this pathway operates within a complex microbial community rather than in a defined-strain colonization setting.

## Conclusions

In conclusion, our study identifies a microbial–host regulatory pathway initiated by *hns*, a single bacterial gene, linking gut microbial metabolism to *rig-5*-dependent dopaminergic vulnerability in *C. elegans* and altered *LSAMP* expression in mammalian systems. By integrating mechanistic, cross-species evidence, we lay the groundwork for microbiome-based precision strategies to stratify and potentially modulate subtype-relevant PD mechanisms through microbial gene function, metabolite production, and downstream host neuronal effectors.

## Supplementary Material

Table_S1_to_3.xlsx Table_S1_to_3.xlsx

Supplementary Methods 0804 V2.docx Supplementary Methods 0804 V2.docx

## Data Availability

The datasets analyzed in this study are available under the following accession numbers: PRJNA1043247 (fecal metagenomics), GSE114160 (*C. elegans* RNA-seq), and GSE334892 (SH-SY5Y RNA-seq generated in this study). Additional data are available from the corresponding author upon reasonable request.
