# Supplementary material for: Escherichia coli hns and methylglyoxal link gut microbial metabolism to host neuronal gene regulation in Parkinson’s disease
Source: Gut Microbes. 2026 Oct 15;18(1):2739674. doi: 10.1080/19490976.2026.2739674 (PMC13679328; doi:10.1080/19490976.2026.2739674)
Supplement: Supplementary file 1 [file KGMI_A_2739674_SM6711.docx]

**Supplementary Methods**

**Sensitivity analyses using alternative subtype definitions**

To assess whether the observed differences in *hns* abundance were robust to alternative operational definitions of PD subtype, we repeated the analyses using each component of the dual-marker framework separately. In the RBD-based analysis, patients with premotor RBD were compared with those with postmotor RBD or no RBD. In the MIBG-based analysis, patients with an abnormal delayed H/M ratio (<1.70) were compared with those with a normal delayed H/M ratio (≥1.70). In both analyses, *hns* abundance in each PD group was also compared with that in healthy controls.

***E. coli* strains**

For bacteria feeding experiments, BW25113 was used as the wild-type (WT) *E. coli*, while *Δhns, ΔmgsA, Δdld, ΔlldD, and ΔaldA E. coli* mutants were obtained from the *E. coli* single-gene deletion mutant library (Keio collection), in which all mutants are generated in the BW25113 background.^1^

**Lifespan assay in *C. elegans***

All lifespan assays were conducted at 20°C. Synchronized L1 larvae were plated onto NGM plates with approximately 40–50 worms per condition. Worm viability was assessed every 2–3 days by gently tapping them with a platinum wire, and worms that died due to vulval bursting were excluded from the analysis. Each lifespan experiment was repeated at least three times, and a representative trial was included Supplementary Figure S3. Statistical analyses and p-values were determined using the log-rank (Mantel-Cox) test, performed with the OASIS application (http://sbi.postech.ac.kr/oasis2/).^2^ For lifespan assays involving MG supplementation, worms were transferred to fresh plates every three days, and methylglyoxal-containing plates were freshly prepared on the day of each transfer.

**Thrashing assay in *C. elegans***

Synchronized worms were maintained under condition-specific settings at 20°C until they reached day 3. To remove residual bacteria, worms were transferred onto empty NGM plates and left for 10 minutes. Approximately 50 worms were then placed into 1 mL of M9 buffer in a 24-well plate. After allowing the worms to settle for 1 minute, videos were recorded using an SMZ745T stereo microscope equipped with an HDMI camera. For subsequent behavioral analysis, the number of thrashes occurring within 30 seconds was manually counted in a randomly selected subset of 25 individuals that exhibited spontaneous locomotor activity at the initiation of the assay.

**Bioinformatics reanalysis of public *C. elegans* bulk RNA sequencing data**

To identify *C. elegans* genes upregulated by *Δhns E. coli*, previously published RNA-seq data from GSE114160 were reanalyzed. Raw read counts from two samples of BW25113-fed worms and two samples of *Δhns E. coli*-fed worms were used for DEG analysis. The ‘DESeq2’ package in R was used to identify DEGs, and GSEA was performed using the ‘ClusterProfiler’ package in R. To examine the expression of *rig-5* orthologs in the SN of PD patients, the mean TPM values of healthy controls and PD patients from GSE148434 were compared. For the striatum, the mean TPM values of healthy controls and PD patients from GSE205450 were analyzed. Mammalian orthologs of *rig-5* were identified using the HGNC Comparison of Orthology Predictions (HCOP) tool ([https://www.genenames.org/tools/hcop/).^3^](https://www.genenames.org/tools/hcop/).3)

**Mouse plasma MG measurement by LC-MS/MS**

Mouse plasma MG was quantified by isotope-dilution LC-MS/MS following derivatization with o-phenylenediamine (OPD), with minor modifications to previously reported methods.^4,5^ Plasma samples were spiked with ¹³C₃-MG as an internal standard and deproteinized with perchloric acid. After centrifugation, the supernatants were reacted with OPD in HCl-DETAPAC solution containing sodium azide for 1 h at room temperature in the dark to form 2-methylquinoxaline (2-MQ). Calibration standards and quality control samples were prepared in 1% bovine serum albumin. MG was quantified using linear calibration curves generated with authentic MG and ¹³C₃-MG standards. Samples were analyzed using an Agilent 1290 Infinity II LC system coupled to a SCIEX QTRAP 5500+ tandem mass spectrometer in positive electrospray ionization mode. Separation was performed on a ZORBAX Eclipse C18 column (3.0 × 100 mm, 3.5 µm) using 0.1% formic acid in water and acetonitrile (60:40, v/v) at 0.35 mL/min. MRM transitions were m/z 145.1 → 77.1 for 2-MQ and m/z 148.1 → 77.1 for ¹³C₃-2-MQ.

**Figure S1. Enrichment of *E. coli* *hns* in Parkinson’s disease is maintained across alternative subtype definitions.**

(A, B) *hns* abundance (RPKM) in the fecal shotgun metagenomic cohort (36 PD and 36 healthy controls), re-analyzed under single-marker subtype definitions instead of the combined premotor RBD–cardiac MIBG criteria used in the main analysis.

(A) Under the premotor RBD–only definition, patients with PD were classified as premotor RBD (n = 16) or premotor non-RBD (n = 20) and compared with healthy controls (HC, n = 36). Both PD subgroups showed significantly higher *hns* abundance than HC, with a numerically higher mean in the premotor-RBD group.

(B) Under the cardiac MIBG–only definition, patients with PD were classified as MIBG-low (n = 26) or MIBG-high (n = 10) and compared with HC (n = 36). Both PD subgroups again showed significantly higher *hns* abundance than HC, with a numerically higher mean in the MIBG-low group.

Data are presented as mean ± SEM. Statistical significance versus healthy controls was determined using the Mann–Whitney U test with Bonferroni correction for multiple comparisons. ***adjusted P < 0.001.

**Figure S2. Impact of various MG metabolic gene deletion mutants on dopaminergic neuroprotection.**

(A–D) Representative fluorescence images (left) and quantification (right) of dopaminergic neurons in the αSyn expressing UA44 strain. UA44 were fed with WT *E. coli*, *Δhns E. coli*, or various MG metabolic gene deletion mutants including *ΔmgsA* (A), *Δdld* (B), *ΔlldD* (C), and *ΔaldA* (D). Quantification includes the number of normal CEP neurites, CEP cell bodies, ADE neurites, and ADE cell bodies in worms under each feeding condition. Data are presented as mean ± SE. Statistical significance was determined using Student’s t-test. *P < 0.05, **P < 0.01, ***P < 0.001; ns, non-significant. n = 30 worms per group.

**Figure S3. Lifespan and locomotor assays of *C. elegans* PD models**

(A–D) MG supplementation experiments using αSyn overexpressing or 6-OHDA-treated PD models.

(A) Lifespan curves of UA44 worms (*dat-1p*::αSyn; *dat-1p*::GFP) fed WT or Δ*hns* *E. coli* ± 10 mM MG.

(B) Thrashing frequency of the same strain under the same conditions as in (A).

(C) Lifespan curves of BZ555 worms (*dat-1p*::GFP) treated with 6-OHDA and fed either WT or Δ*hns* *E. coli*, with or without 10 mM MG.

(D) Thrashing frequency of the same strain under the same conditions as in (C).

(E) Statistical data corresponding to lifespan assays in Figures S3A, and S3C. The table presents data from MG supplementation experiments, including the number of worms, mean lifespan ± SEM, percent change relative to control, and p-values from log-rank tests.

(F–J) *rig-5* RNAi experiments using neuronally RNAi-competent *C. elegans* PD models.

(F) Lifespan curves of neuronally RNAi-competent UA44 worms (UA44; TU3401) fed WT or Δ*hns* *E. coli* with or without *rig-5* RNAi.

(G) Thrashing frequency of the same strain under the same conditions as in (F).

(H) Lifespan curves of neuronally RNAi-competent BZ555 worms (BZ555; TU3401) treated with 6-OHDA and fed WT or Δ*hns* *E. coli* with or without *rig-5* RNAi.

(I) Thrashing frequency of the same strain under the same conditions as in (H).

(J) Statistical data corresponding to lifespan assays in Figures S3F, and S3H. The table presents data from *rig-5* RNAi experiments in neuronally RNAi-competent PD models, including the number of worms, mean lifespan ± SEM, percent change relative to control, and p-values from log-rank tests.

Data from locomotor assays are presented as mean ± SEM. Statistical significance was assessed using Student’s *t*-test. *P < 0.05, **P < 0.01.

**Figure S4. Up-regulated genes by *Δhns* *E. coli* in bulk RNA-seq analysis**

(A) Gene set enrichment analysis (GSEA) of cellular components in *C. elegans* fed with *Δhns* *E. coli* compared to WT *E. coli*. Among the enriched categories, "Synapse" and "Neuron projection" were selected for further analysis due to their relevance to neuronal structure and function.

(B) Expression levels of genes categorized under the Gene Ontology (GO) term “Neuron projection” in *C. elegans* fed with Δ*hns* *E. coli* versus WT *E. coli*.

(C) Expression levels of genes associated with the GO term “Synapse” under the same conditions.

(D) Expression levels of genes annotated in both “Neuron projection” and “Synapse” categories. The data suggest that *Δhns* *E. coli* feeding enhances neuronal connectivity-related gene expression. Data are presented as mean ± SE. Statistical significance was determined using Student’s *t*-test for group comparisons. *P < 0.05, **P < 0.01.

**Figure S5. Expression patterns of *rig-5* orthologs in human neuronal cells and mouse brain.**

(A) Predicted human orthologs of *C. elegans rig-5*, identified using the HCOP (HGNC Comparison of Orthology Predictions) tool provided by the HUGO Gene Nomenclature Committee (HGNC). The table lists 10 candidate orthologs, including their corresponding WormBase Gene IDs, HGNC symbols, Ensembl Gene IDs, NCBI Gene IDs, and supporting orthology resources (Ensembl, Panther, OrthoDB). *SPEGNB* was not included in subsequent experiments and analyses due to its negligible expression in brain tissue.

(B) Relative mRNA levels of *IGSF5* in SH-SY5Y cells treated with increasing concentrations of MG (0.125–1 mM) alone (red) or in combination with 10 mM NAC (blue). Data are presented as mean ± SEM. Statistical significance was determined using Student’s t-test. *P < 0.05, **P < 0.01.

(C) Relative mRNA levels of *Lsamp* measured across various brain regions: SN, striatum (STR), hippocampus (HIP), cortex (CTX), prefrontal cortex (PFC), and olfactory bulb (OB). Data are presented as mean ± SEM. Statistical significance was determined using Student’s t-test. **P < 0.01.

**Figure S6. Reduced plasma MG in mice colonized with *Δhns* *E. coli*.**

(A) Quantification of plasma MG (ng/mg protein). Plasma MG was significantly lower in mice colonized with *Δhns* *E. coli* than in those colonized with WT *E. coli*.

(B, C) Representative LC-MS/MS chromatograms of the MG-derived 2-methylquinoxaline (2-MQ; arrow) in plasma from mice colonized with WT *E. coli* (B) or *Δhns* *E. coli* (C). In the chromatograms, the blue trace indicates the analyte (2-MQ), and the pink trace indicates the internal standard (IS, ¹³C₃-2-MQ).

Data are presented as mean ± SEM. Statistical significance was determined using Student’s t-test. *P < 0.05. n = 3 per group.

**Supplementary References**

1. Baba T, Ara T, Hasegawa M, et al. Construction of Escherichia coli K-12 in-frame, single-gene knockout mutants: the Keio collection. Mol Syst Biol 2006;2:2006 0008.

2. Han SK, Lee D, Lee H, et al. OASIS 2: online application for survival analysis 2 with features for the analysis of maximal lifespan and healthspan in aging research. Oncotarget 2016;7:56147-56152.

3. Yates B, Gray KA, Jones TEM, Bruford EA. Updates to HCOP: the HGNC comparison of orthology predictions tool. Brief Bioinform 2021;22.

4. Rabbani N, Thornalley PJ. Measurement of methylglyoxal by stable isotopic dilution analysis LC-MS/MS with corroborative prediction in physiological samples. Nat Protoc 2014;9:1969-1979.

5. Scheijen JLJM, Schalkwijk CG. Quantification of glyoxal, methylglyoxal and 3-deoxyglucosone in blood and plasma by ultra performance liquid chromatography tandem mass spectrometry: evaluation of blood specimen. Clin Chem Lab Med 2014;52:85-91.
